# A History of the Pharmacological Treatment of Bipolar Disorder

**DOI:** 10.3390/ijms19072143

**Published:** 2018-07-23

**Authors:** Francisco López-Muñoz, Winston W. Shen, Pilar D’Ocon, Alejandro Romero, Cecilio Álamo

**Affiliations:** 1 Faculty of Health Sciences, University Camilo José Cela, C/Castillo de Alarcón 49, 28692 Villanueva de la Cañada, Madrid, Spain; 2 Neuropsychopharmacology Unit, Hospital 12 de Octubre Research Institute (i+12), Avda. Córdoba, s/n, 28041 Madrid, Spain; 3 Portucalense Institute of Neuropsychology and Cognitive and Behavioural Neurosciences (INPP), Portucalense University, R. Dr. António Bernardino de Almeida 541, 4200-072 Porto, Portugal; 4 Thematic Network for Cooperative Health Research (RETICS), Addictive Disorders Network, Health Institute Carlos III, MICINN and FEDER, 28029 Madrid, Spain; 5 Departments of Psychiatry, Wan Fang Medical Center and School of Medicine, Taipei Medical University, 111 Hsin Long Road Section 3, Taipei 116, Taiwan; shenwinw@gmail.com; 6 Department of Pharmacology, Faculty of Pharmacy, University of Valencia, Avda. Vicente Andrés, s/n, 46100 Burjassot, Valencia, Spain; m.pilar.docon@uv.es; 7 Department of Pharmacology and Toxicology, Faculty of Veterinary Medicine, Complutense University, Avda. Puerta de Hierro, s/n, 28040 Madrid, Spain; manarome@ucm.es; 8 Department of Biomedical Sciences (Pharmacology Area), Faculty of Medicine and Health Sciences, University of Alcalá, Crta. de Madrid-Barcelona, Km. 33,600, 28871 Alcalá de Henares, Madrid, Spain; cecilioalamo@hotmail.com

**Keywords:** bipolar disorder, pharmacological treatment, mood stabilizer drugs, lithium, antiepileptic drugs, antipsychotic drugs

## Abstract

In this paper, the authors review the history of the pharmacological treatment of bipolar disorder, from the first nonspecific sedative agents introduced in the 19th and early 20th century, such as solanaceae alkaloids, bromides and barbiturates, to John Cade’s experiments with lithium and the beginning of the so-called “Psychopharmacological Revolution” in the 1950s. We also describe the clinical studies and development processes, enabling the therapeutic introduction of pharmacological agents currently available for the treatment of bipolar disorder in its different phases and manifestations. Those drugs include lithium salts, valproic acid, carbamazepine, new antiepileptic drugs, basically lamotrigine and atypical antipsychotic agents (olanzapine, risperidone, quetiapine, ziprasidone, aripiprazole, asenapine, cariprazine and lurasidone). Finally, the socio-sanitary implications derived from the clinical introduction of these drugs are also discussed.

## 1. Introduction

Bipolar disorder is a mental pathology that has been known since ancient times and has its origin in the concept of “mania,” a term that comes from the Greek “*μανια*,” meaning “madness” or “frenzy” [1]. The father of medicine, Hippocrates, already qualified mania as a subtype of mental illness. The term can even be found in the early Ancient Greece literature, as in Homer’s *Iliad*, referring to the uncontrolled anger of Achilles against Agamemnon [2]. Philippe Pinel [3] established a series of categories of this type of madness (mania with delirious and non-delirious states) and throughout the19th century this entity was closely linked to psychosis. Halfway through the 19th century, with the rise of the anatomoclinic mentality, somaticist movements began to emerge in the field of psychiatry. In this context, Jacques-Joseph Moreau de Tours and Wilhelm Griesinger postulated a structural alteration of the brain as the etiological basis of “madness” [4]. Finally, in this anatomoclinical atmosphere, Emil Kraepelin, at the end of the 19th century coined the concept of manic-depressive psychosis and dissociates this disorder from the so-called dementia praecox (schizophrenia) [5]. In the 1950s, Karl Leonhard first introduced the concept of polarity in understanding affective disorders [6], which would later be included among the DSM diagnostic criteria in *Diagnostic and Statistical Manual of Mental Disorders* of the American Psychiatric Association (APA). Accordingly, there would be unipolar patients, who only experience depressive episodes and bipolar patients, presenting a manic or hypomanic episode [7].

Until relatively recent dates, bipolar disorder had not received properly deserved scientific and social recognition as it enjoys today [8]. Due to “scientific negligence,” compared to other psychiatric disorders—for example, major depression or schizophrenia—the lack of knowledge about its etiopathogenesis, the erroneous underestimation of its prevalence and the status of therapeutic pseudo-orphanage had not been amended until almost two decades ago [9]. In fact, until the mid-1990s, before valproate’s anti-manic efficacy of was demonstrated, the only drug available for treating those patients was lithium salts.

From a current scientific research perspective, bipolar disorder is an emerging pathology [9]. From a clinical perspective, it presents an important morbidity/mortality [10]. The well-known epidemiological studies Epidemiological Catchment Area Study (ECA) in 1981 and National Comorbidity Survey (NCS) in 1991 estimated the prevalence of bipolar disorder in about 1% of the population and it is currently estimated that its prevalence throughout the lifespan is between 1% and 2% of the population over 20 years of age [11]. It is a serious mood disorder of a chronic nature, characterized by the constant or irregular appearance of alternative episodes of mania/hypomania with euthymia/depressive episodes [12]. Traditionally, a difference has been made between bipolar disorder type I, which is characterized by the presence of, at least, a manic or mixed episode in combination with depression; and bipolar disorder type II, characterized by one or more depressive episodes, with at least one episode of hypomania lasting longer than four days, with bipolar disorder type II being more frequent than type I. Cyclothymic disorder has a recurrent and chronic course, with greater symptomatic oscillation but of less severity than that observed in bipolar disorder types I and II. Between 15–50% of cyclothymic patients usually progress to either bipolar disorder type I or, mainly, to type II [13]. Nevertheless, nowadays a tendency exists to consider this pathology from a broader context, including all those cases with less obvious and apparent clinical manifestations. This way of approaching the disease, called “bipolar spectrum,” implies prevalence ranging from 2.8% to 6.5% of the population [10,14,15,16].

Bipolar disorder, largely due to its chronic and recurrent course, poses an important burden for the patient, the family and the society and its treatment is essential to avoid the main complications of the disease. In developed countries, bipolar disorder is ranked among the top 10 causes of disability [17]. According to the World Health Organization (WHO), bipolar disorder is the fourth cause of neuropsychiatric disability in people aging 15–44 years. Additionally, the use of health resources that bipolar patients make is greater than that of patients with depressive or chronic medical conditions [18]. One should also bear in mind that the most serious complication of bipolar disorder is suicide. Between 25–50% of patients with bipolar disorder attempt suicide, with prevalence of 32.4% and 36.3% for bipolar disorder type I and type II, respectively [19]. It is in the depressive phase that most attempts and completed suicides occur. Other important burdens associated with bipolar disorder are increased mortality from medical causes, substance abuse (mainly alcoholism), comorbidity with other psychiatric disorders such as anxiety, behaviour and eating disorders and chronic affective symptoms. To note, bipolar disorder patients have enormous economic burden of this disorder, estimated in the USA to be about $31 billion in direct costs and $120 billion in indirect costs [20].

Until the discovery of lithium’s antimanic effect, the pharmacological tools used in clinical practice had limited efficacy, not only in this type of patient but in the entire field of mental pathology [21]. This was in part a result of the inheritance and influence of Benedict Agustin Morel, a degenerationist who considered that the cause of mental disorders was hereditary transmission, implying their incurability [4]. Yet, during the first half of the 20th century, the first drugs used systematically in the treatment of manic patients were introduced, with barbiturates standing out among them. The antimanic efficacy of lithium salts was confirmed during the 1950s and in the late 1960s it was finally demonstrated in Europe that lithium was effective in the prophylaxis of manic-depressive episodes in bipolar disorder [22]. But the Food and Drugs Administration (FDA) of the United States resisted to authorize this drug for its antimanic indication until 1970 [23] and for its indication as maintenance/prophylactic treatment of manic-depressive episodes until 1978. Twenty-eight years later, in 1995, valproic acid, an anticonvulsant agent, was approved by the FDA for its antimanic indication. In parallel to valproic acid, carbamazepine, another antiepileptic drug, was developed for the treatment of patients with bipolar disorder. This drug has been authorized by numerous regulatory agencies in multiple countries and enjoys a good acceptance by psychiatrists worldwide. Finally, since 2000, different atypical antipsychotic drugs (AADs) have been authorized by the FDA for their antimanic (olanzapine, risperidone, quetiapine, ziprasidone, aripiprazole, etc.) or antidepressant indication (quetiapine, lurasidone). Lamotrigine, a modern antiepileptic agent, has been authorized for the prevention of depressive episodes in bipolar disorder. Table 1 shows the most important historical milestones in the pharmacological treatment of bipolar disorder. Overall, the pharmacological treatment of bipolar disorder is complex and treatment guidelines exist for its different phases—manic episodes, depressive episodes and maintenance (or prophylaxis) periods. In this sense, polypharmacy combining drugs from different therapeutic groups and different mechanisms of action is the norm rather than the exception.

## 2. The Therapeutic Precedents of the Psychopharmacological Era

**Figure 1:**
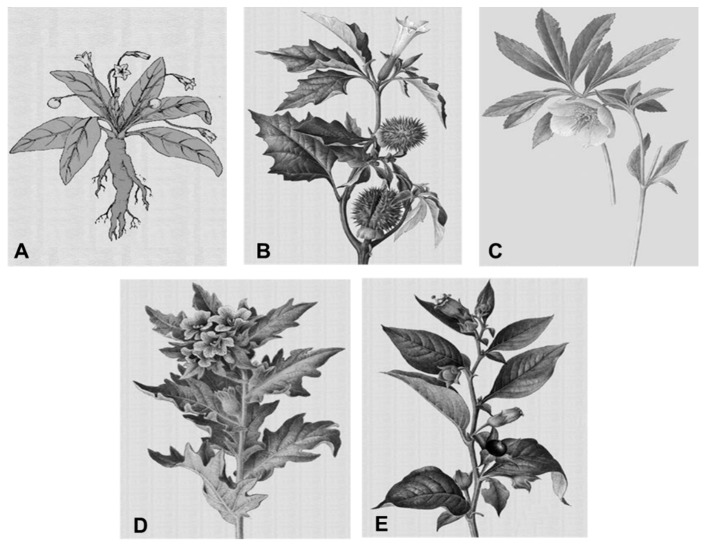
Illustrations of the most relevant plants in the *Solanaceae* family, from which several alkaloids were obtained to be used in psychiatric practice as sedatives prior to modern psychopharmacology: (**A**) Mandrake (*Mandragora officinalis*); (**B**) Stramonium (*Datura estramonio*); (**C**) Eléboro (*Helleborus niger*); (**D**) Hellebore (*Hyosciamus niger*); (**E**) Belladonna (*Atropa belladonna*).

Until the clinical introduction of lithium salts, sedatives [24] were the main axis of pharmacological treatment of manic symptoms. During the second half of the 19th century, a time referred to by some authors as the “alkaloid period” [25], those agents were the most used sedatives. They were introduced into psychiatric care thanks to the isolation of morphine from opium in 1805 by Friedrich Wilhelm Sertürner [26]. In the book *Traité des Maladies Mentalis*, published in 1865, Griesinger noted that anxious and agitated patients improve after receiving opium [27]. Hashish was also used as a sedative and its effects were studied in depth by Moreau de Tours [28]. But the most successful alkaloids in psychiatry were isolated essential chemicals of various components of the *Solanaceae* family (Figure 1), the plants known for their hallucinogenic effects. One of them was hyoscyamus, which was isolated in 1839 by chemists from the company E. Merck (Darmstadt, Germany) to show their sedative and hypnotic properties as described in 1868 by the Viennese pharmacologist Karl Schroff. Hyoscyamine was another alkaloid that became a popular ingredient in many cocktails in the latest years of the 19th century. Finally, in 1880, another hyoscyamine alkaloid—hyoscine—isolated by Albert Ladenburg in Germany. Hyoscine (called scopolamine in North America) was then also a widely used ingredient in psychiatric cocktails (such as the famous Hyoscine CoA, containing hyoscine, morphine and atropine), was often used in controlling highly excited and aggressive manic patients [29,30].

The advent of synthetic chemistry was another of the great milestones in pharmacology in the 19th century, because for the first time in history, synthesized substances instead of existing nature plants with biological activity were produced [31]. Choral hydrate was first produced psychopharmaceutically [32]. Synthesized in 1832 by a chemist from Giessen, Justus von Liebig, it was not assessed as a hypnotic until 1869, by the pharmacologist Mathias Otto Liebreich. The following year, the American psychiatrist William J. Elstun reported that 5 patients (3 manic and 2 melancholic) from Indiana Hospital for the Insane improve after receiving chloral hydrate [33]. Soon chloral hydrate was replacing both morphine and solanaceous alkaloids because it could be conveniently given orally. Among its great contributions to the history of psychopharmacology, chloral hydrate was the first drug to make home treatment possible, without the need to have a patient admitted to a psychiatric institution [25].

**Figure 2:**
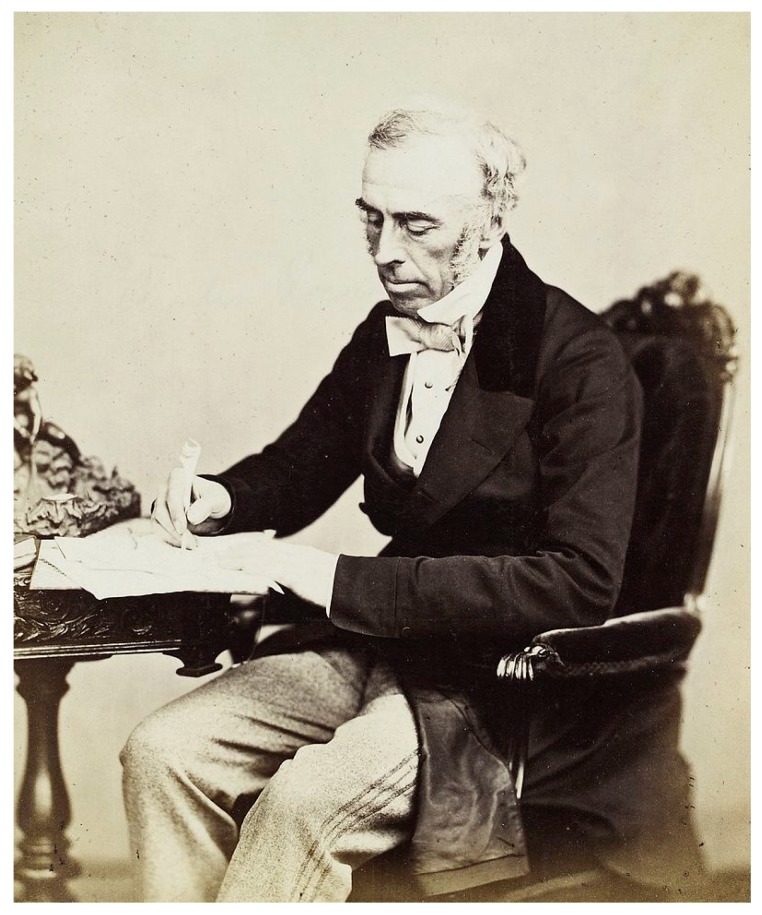
Portrait of Sir Charles Locock (1799–1875) dated 1862. Locock was an obstetrician, First Physician Accoucheur to the Queen Victoria and a member of the St. Albans Medical Club.

Bromides were another therapeutic contribution in the 19th century [32]. Isolated in 1826 by the pharmacist from Montpellier, Antoine Balard, bromine was used as an iodine substitute as a food additive. It became popular in France, where they could already observe that bromine salt-induced sedation (“*invresse bromurique*”). Although first used as antiepileptics, bromides in the second half of the 19th century were widely used as sedatives for this indication in 1857 by the London internist and Queen Victoria’s obstetrician, Sir Charles Locock (Figure 2). Similarly, they were also used as one of the best remedies for controlling agitation in psychotic, manic and melancholic patients, as well as those affected by different phobias, which were favourably used in European asylums and mental hospitals. The use of bromides had extended until the early years of the 20th century [34].

By the end of the century, new drugs with sedative activity were added to this therapeutic arsenal. Those drugs—codeine, papaverine, chloroform potion, chloral syrup, paraldehyde, carbamic acid, or hydrocyanic acid—were isolated in pharmacology laboratories. Even a treatment with adrenal cortex extracts was suggested, following the observation that serum cholesterol levels were increased in bipolar patients [35,36]. Subsequently, the introduction of some nonspecific chemical preparations included succinic dinitrile, malonic nitrite, or lactic acid, although delivering unsatisfactory results [37]. Another commonly used drug was sodium diphenylhydantoin (Dilantin^®^), tested by Kalinowsky and Putnam [38] but producing only limited success in cases of manic excitement.

**Figure 3:**
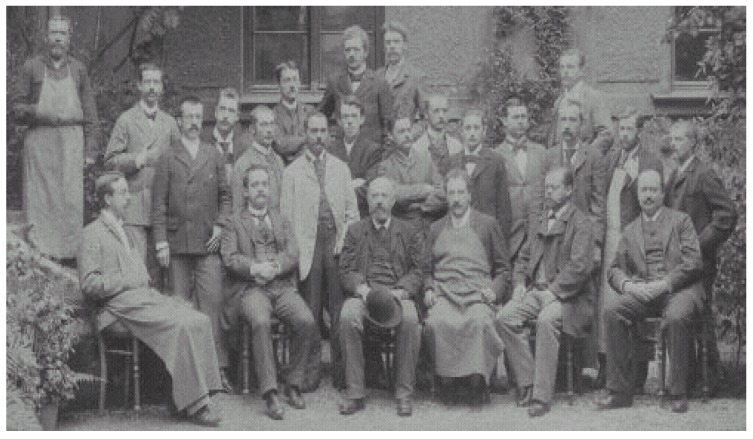
Adolf von Baeyer (1835–1917), discoverer of barbituric acid, in the centre with a hat in his hands, together with his team from the Chemistry Laboratory of the Munich Academy of Sciences, in 1893.

Without a doubt, barbiturates had been the most widely used agents in treating manic patients during the first half of the 20th century and until the mid-1950s [39,40]. Chemically, those uranium compounds have a six-element ring structure, with a central nucleus of malonylurea. They were synthesized in 1863 by Adolf von Baeyer (Figure 3). Barbital (diethyl-barbituric acid) was the first agent of this family introduced clinically as a hypnotic in Germany. It was marketed in 1903 by the company Bayer & Comp. (Elberfeld, Germany) under the name Veronal^®^ (Emil Fischer and Joseph von Mehring), although it had been synthesized long before (1882) by Max Konrad and Max Guthzeit. This drug was presented itself as an anticonvulsant, then a sedative and, eventually a hypnotic. Veronal^®^ was found to calm manic patients, restore sleep in melancholic patients and induce sleep in patients with insomnia [39,40]. Due to changes in chemical structure of the barbituric acid molecule, more than 2500 different barbiturates were synthesized but only about 50 of them have ever been on the market. One of the first such drugs and perhaps the most used later, was phenobarbital, marketed in 1912 by the company Bayer under the name of Luminal^®^. This long-acting drug, was widely used as an antimanic, anxiolytic and anticonvulsant; other barbiturates with short and intermediate action (such as secobarbital, amobarbital, pentobarbital, etc.) were initially used as hypnotics [39].

**Figure 4:**
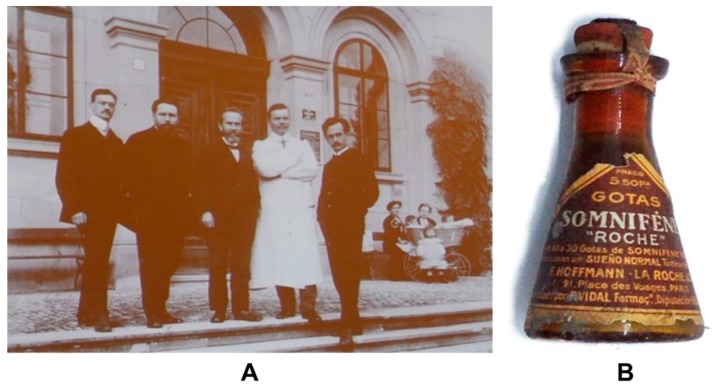
Jakob Klaesi (right) (1883–1980) with the Burghölzli Hospital psychiatric staff, including its director, Eugen Bleuler (centre), in a photograph taken around 1910 (**A**) and a container of Somnifen drops of the first third of the century 20th century, commercialized in Spain by F. Hoffmann-La Roche & Cie, Paris (**B**).

The hypnotic properties of some barbiturates were quickly used in treating psychotic and manic patients, as they induced a deep and prolonged sleep state [41]. The pioneer in the use of these techniques was the Italian psychiatrist Giuseppe Epifanio, assistant at the University Psychiatric Clinic of Turin. Published in 1915 in an Italian journal in the middle of World War I, this article did not get proper attention from the international scientific community [42]. The clinical introduction of those techniques is attributed historically to Jakob Klaesi, a psychiatrist at the University Clinic of Zurich (Figure 4A). Klaesi enjoyed great prestige at the time right after he proposed his sleep cures in 1920 [43]. These techniques consisted of premedication with morphine and scopolamine, as well as the subsequent use of so-called Somnifen^®^, administered intravenously or subcutaneously (Figure 4B). That was a mixture of diethyl and dipropenyl-barbituric acid, prepared by the Swiss firm Hoffmann-LaRoche (Basel) in a process lasting at least 6–7 days. Another preparation of these characteristics was the so-called Cloetta or Cloettal mixture [44,45], using barbiturates rectally, amyl hydrate, alcohol, chloral hydrate, digitaline, paraldehyde and ephedrine. The main clinical indications of “sleep cures” were manic excitations and agitated depressions, although they were also tested in schizophrenia with less satisfactory results [41].

## 3. Lithium as an Antimanic Agent

Lithium is coined from the Greek “lithos,” meaning stone. Chemically, lithium is the simplest drug among those used in psychiatric therapy, as it is the lightest metal in nature. The distribution of this metal in small quantities is wide, being detected in seawater, the water of rivers and springs, the organs of different animals, the remains of many plants and even in meteorites [46].

**Figure 5:**
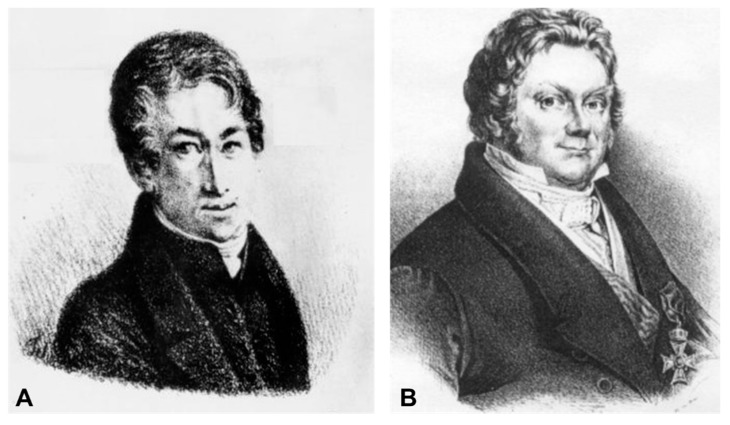
Mid-nineteenth century lithographs of the two Swedish chemists responsible for the discovery of lithium: Johann August Arfwedson (1792–1841) (**A**) and Jöns Jakob Berzelius (1779–1848) (**B**), Professor of Chemistry and Pharmacy at the Karolinska Institute. Both were members of the Royal Swedish Academy of Sciences.

Lithium and mental disorders enjoy a long story. Although the great Greek, Roman and Arab classical authors did not specifically mention lithium in their medical works, the use of alkaline waters for the treatment of the mental illness known as “mania” dates from the time of Caelius Aurelianus, in the 5th century. In a section dedicated to the treatment of mania, this author wrote, “*utendum quoque naturalis aquis, ut sunt nitrosae*” (“natural waters, such as those of alkaline springs, must be used”). In 1817, this alkaline metal was isolated from a mineral known as petalite by the Swedes Johann August Arfwedson (Figure 5A) and Jöns Jakob Berzelius (Figure 5B) and they originally named it lithium. Since lithium has been incorporated into therapeutics, being used in treating different pathologies, both organic (gout and cancer, for example) and neurological (epilepsy) [47,48].

**Figure 6:**
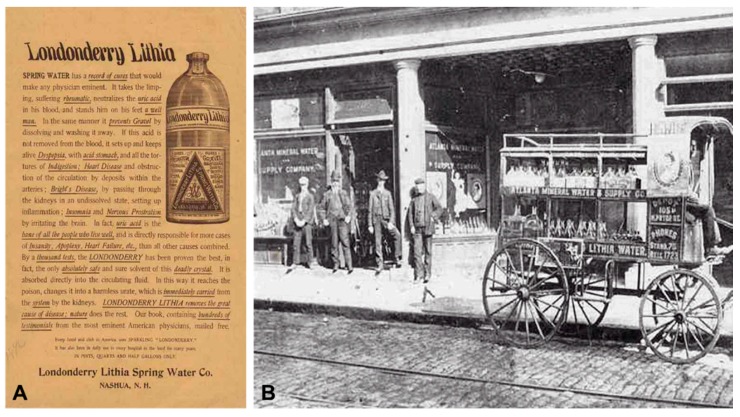
Two examples of the popularity of lithiated waters at the end of the 19th century: a commercial of Londonderry Lithia (Londonderry Lithia Spring Water and Co., Nashua, NH, USA), showing all its supposed therapeutic properties (**A**) and a photograph of the late 19th century of the head office and distribution system of Atlanta Mineral Water and Supply Co. (Atlanta, GA, USA) (**B**), advertising its Lithia Water.

In addition, one should consider the great popularity that, around the years of the turn of the century, the “intake of waters” gained, mainly in the USA. Thus, a series of products that supposedly contained lithium water were commercialized, using aggressive advertising stunts. Such is the case of Buffalo Lithia Water (“the best natural remedy for excess uric acid in blood”), Tedyffrin Water (“cures dyspepsia, rheumatism, Bright’s disease and dissolves bladder stones”), or Manadnock Lithia Spring Water (“the most wonderful spring water known in the world”). Even a beer manufactured with lithium water (Lithia Beer), was marketed in Wisconsin and it was highly appreciated in those decades (Figure 6). All of those stated in their instructions that they were harmless and effective remedies for treating all kinds of nervous disorders [47]. But the decline in consuming lithium waters and, therefore, the popularity of lithium, took place at the end of the first decade of the 20th century, as the public came to know that these products only contained spectroscopically traces of lithium and, above all, due to the first cases of intoxication of cardiac patients, as a consequence of chronic use [49]. In *The Practitioner*, several cases of “cardiac depression and even dilation as a result of an excessive and continued consumption of lithium tablets” were reported in 1907 [50]. In spite of all this, in 1929, Charles Leiper Grigg (Howdy Corporation, St. Louis, MO, USA) invented the 7-UP Lithiated Lemon Soda, with the advertising claim of “an abundance of energy, enthusiasm, clear complexion and shining eyes,” referred to simply as 7-UP since 1936, although lithium was part of its composition until the 1940s, coinciding with the North American cases of toxicity discussed above [51].

**Figure 7:**
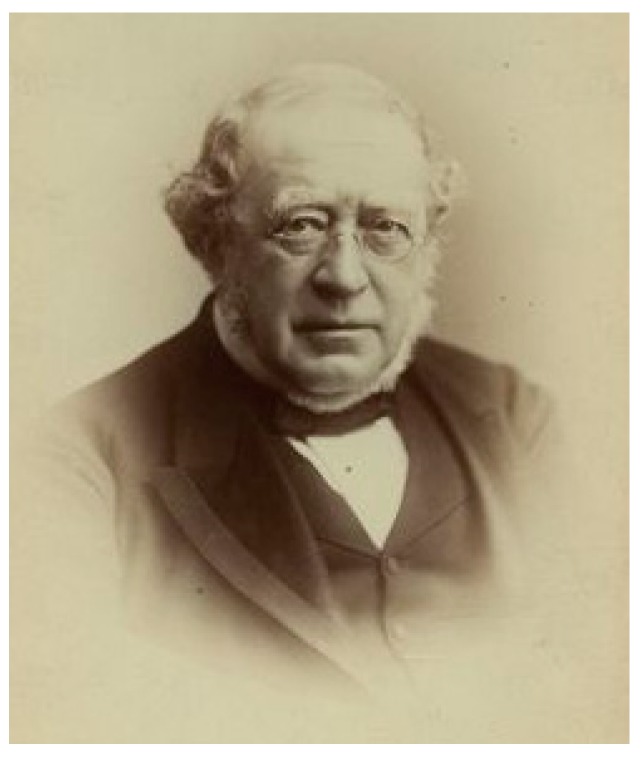
Sir Alfred Baring Garrod (1819–1907), professor of Materia Medica and Therapeutics at King’s College Hospital and President of the Medical Society of London in 1860. He was knighted as “Sir Alfred Baring Garrod” and in 1890 was appointed “Physician Extraordinary” to Queen Victoria.

In the mid-nineteenth century, the rise of the “uric acid diathesis” theory, proposed by Sir Alfred B. Garrod (Figure 7), played an important role in the introduction of lithium into pharmacological therapy [52]. In 1859, Alexander Ure, a surgeon at the Western Ophthalmic Institution (London), could dissolve a series of bladder stones in a lithium carbonate solution [47]. Almost at the same time, Garrod could verify, in the corpses of patients with gout, that when phalanges with deposits of uric acid were introduced in lithium, potassium or sodium carbonate solutions, the quickest dissolution of such deposits occurred in lithium carbonate. This theory was based on the observation that, in test tubes, lithium salts could dissolve urate deposits from cartilage. Hence, lithium was becoming widely used in treating kidney stones, rheumatism and gout [53]. In fact, in the United Kingdom, lithium was sporadically and unofficially used in treating gout until the 1970s [54].

**Figure 8:**
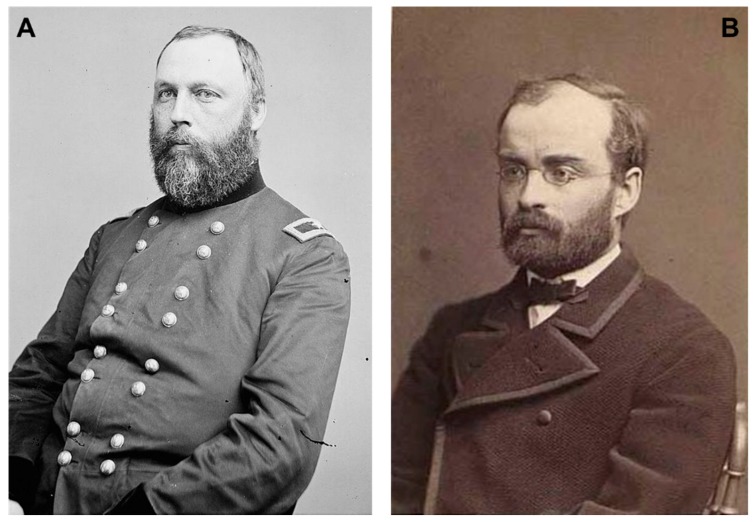
Pioneers in the use of lithium in the treatment of mental disorders: William Alexander Hammond (1828–1900) (**A**), Surgeon General of the United States Army, professor of anatomy and physiology at the University of Maryland School of Medicine, professor of Nervous and Mental Diseases at Bellevue Hospital and at the New York University; and Carl Georg Lange (1834–1900) (**B**), Danish physician who made contributions to the fields of neurology, psychiatry and psychology.

Similarly, some of mental disorders were hypothesized to be related to having high levels of urates, being referred to as “gout that affects the head” and “gouty mania”. Lithium treatments were also recommended in those patients [47]. It even came to be occasionally used in treating other psychiatric disorders. For example, the American general surgeon William Hammond (Bellevue Hospital in New York) (Figure 8A) used lithium bromide in treating acute mania, although he could not discern whether the effects observed were due to bromide or lithium [55]; and the Danish internist Carl G. Lange (University of Copenhagen) (Figure 8B) stated in 1886 that the use of a mixture containing lithium was capable of preventing the so-called “periodic depression” [56]. On the other hand, in the British Isles, as Henderson and Gillespie [57] recalled that lithium waters were believed to have important virtues for treating mental illness. Even much earlier, Sir John Floyer, in his book *History of Cold Bathing* (1722), described that the success achieved in a case of mania with a lithium-based treatment. Still, all these events went completely unnoticed for many years.

The interest in lithium came back in 1927, when the use of lithium bromide in the treatment of epilepsy was postulated. The key contribution was from David M. R. Culbreth, who observed that lithium bromide was the most sedative and hypnotic of all bromides [47]. Yet, it would not be until the end of the 1940s when lithium salts finally became part of the therapy of mental illnesses.

### 3.1. The Experiments of John Cade

**Figure 9:**
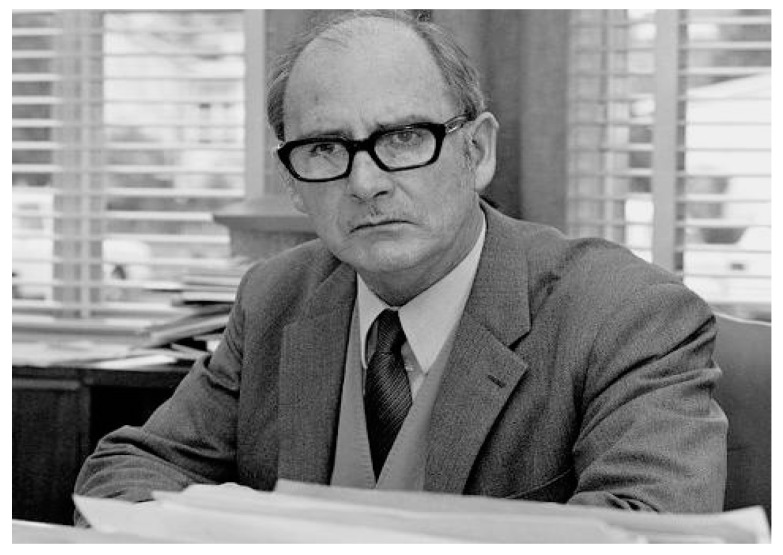
John Frederick Cade (1912–1980), authentic pioneer of modern psychopharmacology with his experiments on lithium.

As in many other events in biomedical research, chance played an important role in the introduction of lithium into the psychopharmacological arsenal [58,59]. Thanks to the careful observation of Australian psychiatrist John F. Cade (Figure 9), who in 1949 was Senior Medical Officer, in the Victorian Department of Mental Hygiene of Australia (Superintendent in the Repatriation Mental Hospital of Bundoora, Victoria, Australia), he noted how patients with thyroid endocrine diseases presented themselves symptoms that were similar to the clinical manifestations of manic-depressive disorder, so that individuals with hyperthyroidism experienced symptoms similar to those seen in individuals in the manic phase, while the traits of thyroid hypofunction were resembling those of the depressive phase [60].

**Figure 10:**
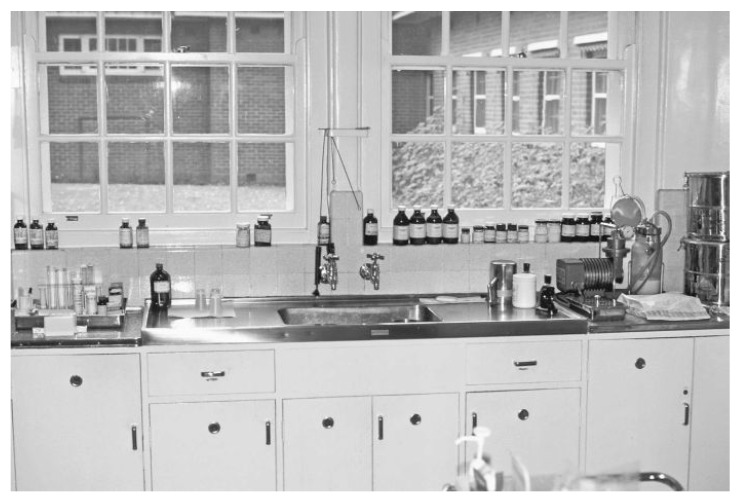
The kitchen at Bundoora Repatriation Mental Hospital where John Cade conducted some of his lithium experiments.

Given this similarity, the Australian psychiatrist questioned whether at the origin of manic-depressive illness is a hormonal dysfunction or some toxin that was being excreted in the urine and so he designed a series of interesting animal research studies (Figure 10). Initially, he collected urine samples from manic and melancholic patients, as well as from healthy controls and after a process of concentration, he injected them intraperitoneally to guinea pigs, at different doses. Some animals which had been treated with high doses of urine suffered convulsive movements, prolonged unconsciousness and even died, which reaffirmed Cade’s idea that the urine of these patients could have contained some sort of toxic substance. At first, he believed that this substance could be urea. But he observed that the urine of manic patients was markedly more toxic than the rest, even when the amount of urea or creatinine was similar between manic and non-manic patients. This study finding made Cade to think that another substance should exist to increase the toxicity of urea, such as uric acid.

**Figure 11:**
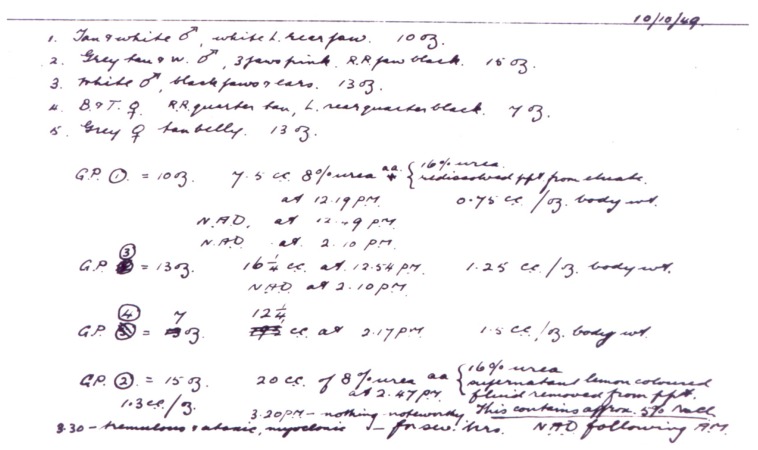
Laboratory notes by Cade regarding the effects of the injection of different compounds and lithium salts to guinea pigs.

To demonstrate this theory, Cade designed a study based on giving laboratory animals of a solution with urea and different concentrations of uric acid (Figure 11). Due to the poor solubility of this substance, Cade turned to lithium urate, a much more soluble salt and surprisingly found that the injection of this salt at 0.5% in the 8% urea solution protected the animals from the previously observed convulsive phenomena, resulting in complete survival. In addition, the animals did not respond to stimuli, not even painful ones and their mobility and appetite for food considerably have been decreased. These observations led Cade to investigate the effects in guinea pigs of the exclusive administration of lithium carbonate. Two hours after having received test drug, animals showed a state of lethargy, which was reversed another two hours later [47,48].

**Figure 12:**
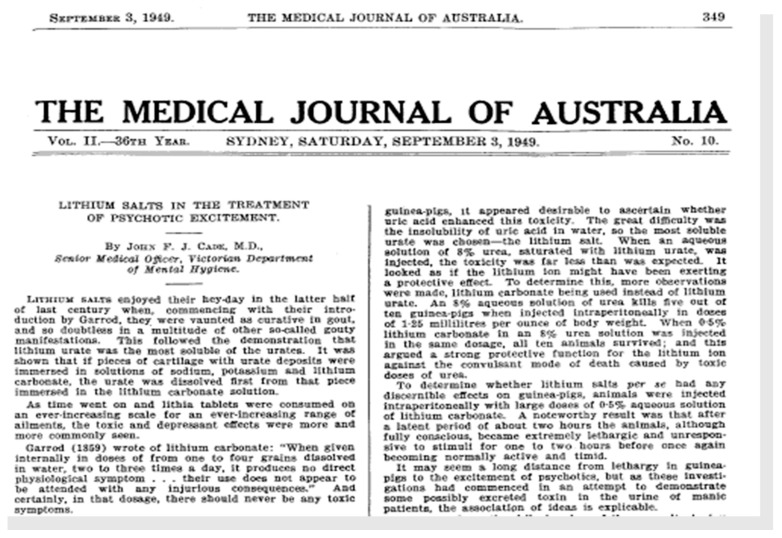
Medical Journal of Australia, Volume 3, September 1949, in which the results of the clinical experiments by John Cade about lithium salts and the handling of hectic manic and schizophrenic patients were published [61].

The results of these studies made Cade to think of the possible benefit that certain manic patients could experience after receiving those lithium salts. But before taking this step and following the great clinicians of the previous century and under the Hippocratic principle of *primum non nocere*, he had lithium citrate self-administered to assess its safety. Immediately after testing the absence of toxicity on himself—which is not illustrative, given his high level of tolerance to suffering after his having spent three years as a prisoner-of-war in the Japanese POW camp of Changi, Singapore, during World War II—he administered, on 29 March 1948, 1200 mg/day of lithium citrate, three times a day in divided dose, to a 54-year-old man, Mr. W. B. in manic excitement of five years of evolution. After a five-days treatment, the patient was evidentially improved. He left the hospital four months later, with a daily ambulatory treatment of 300 mg of lithium carbonate, twice a day, because this salt caused less nausea than citrate. The recovery was so clear that the patient could return to the job before his hospital admission. Cade observed the same results in nine other patients, obtaining the best responses in excited individuals. The effects of lithium were also studied in 6 patients with schizophrenia with agitation and in 3 patients with chronic melancholic depression. The results of these studies were published in 1949, in an article in *The Medical Journal of Australia*, entitled “*Lithium salts in the treatment of psychotic excitement*” (Figure 12) [61]. This publication is considered by many authors as the starting point of the so-called “psychiatric pharmacology revolution” [62,63]. The summaries of this work consisted of the following:

- Lithium salts showed great efficacy in the treatment of manic disorder and they did so in a short period of time within several days.
- Lithium was also relatively effective in treating manic manifestations in early dementia. Three of the six most agitated schizophrenic patients became eased and calm and were docile and treatable for the first time in years. All of them returned to their original state when lithium was stopped.
- The effectiveness of lithium in chronic depression was not shown.
- The recommended dosage regimen was 900 mg/day, to be divided in 300 mg 3 times a day until clinical improvement was observed and 300 mg/day during the maintenance period.
- The discontinuation of lithium treatment led to the reappearance of manic symptoms.
- The adverse effects of lithium therapy have two—digestive system (nausea, vomiting, diarrhoea, abdominal pain, etc.) and nervous system (tremors, dizziness, asthenia, depression, etc.)—categories of side effects, which were disappeared quickly in 2 to 4 days) after lithium discontinuation. To resume treatment, the patient needs to start receiving from a lower dose, or carbonate was to substitute for citrate, since carbonate salt is more soluble and more easily absorbed.

Nevertheless, in spite of the interesting perspectives that Cade’s work posed, lithium salts did not become widely used in psychiatry until the mid-1960s, as a result of a series of events that unfortunately coincided in time. This is the case of the clinical introduction and upsurge of neuroleptic drugs, which set aside the scientific interest in lithium. Also, many fatal poisonings that occurred in the late 1940s in the USA, when those lithium salts were marketed as a substitute for sodium salts in patients with heart disease [48,64,65]. Then, the media contributed to the discrediting of this drug. Additionally, Cade himself stated, “the discovery made by an unknown psychiatrist with no research training in a small chronic hospital with primitive techniques and negligible equipment was not likely to command attention” [66]. Cade abandoned his research with lithium and devoted himself to the study of psychotropic effects of other alkaline metals, such as rubidium and caesium. Cade further recalled that, “…in March 1949, lithium was effectively excommunicated as a therapeutic tool, at least in the United States” [67].

### 3.2. From Discredit to Formal Recognition of the Antimanic Efficacy of Lithium

For its part, Kline [68] lists another series of reasons that prevented the scientific recognition and clinical use of lithium, including the distrust of psychiatrists in such a simple substance, which was meant to treat a disease as complex as bipolar disorder. Also, their lack of understanding of how a single substance could show efficacy against both mania and depression. In addition, he points out the absence of commercial interest that the pharmaceutical industry could have in a natural substance that was cheap to obtain and not patentable. It is also worth noting that some clinical trials, poorly designed from the methodological point of view (in which lithium serum levels had to be monitored for safety reasons) and drew negative results. In any case, the loss of interest in lithium was such that even in the prestigious work Goodman and Gilman’s *The Pharmacological Basis of Therapeutics*, in its 1960 edition, stating “The lithium ion has no therapeutic applications. The only pharmacological interest in lithium lies in the fact that it is a toxic ion” [69].

**Figure 13:**
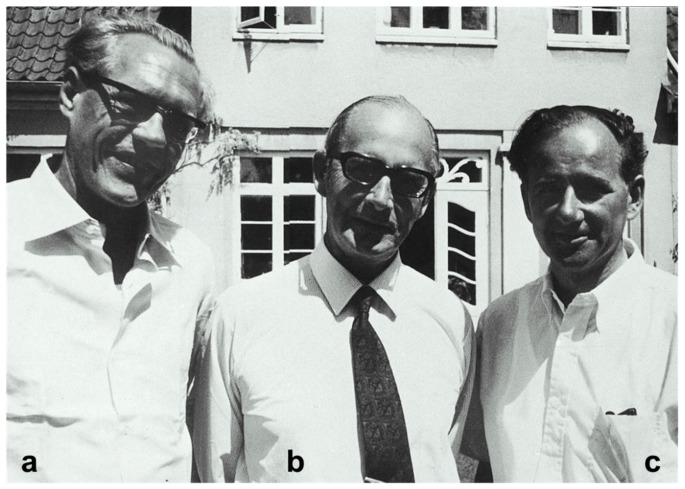
Three great pioneers of the clinical introduction of lithium: Poul Christia Baastrup (**a**), John Cade (**b**) and Mogens Schou (**c**).

Cade’s communication attracted the attention of other Australian researchers who delved into the contributions of this author [70,71]. The Danish psychiatrist, Mogens Schou (Figure 13), stood above all of them. In his family, some members had manic-depressive disorder. He and his team from Aarhus, Denmark—mainly his colleague Poul Christian Baastrup (Figure 13)—started the first randomized clinical trials using this drug in the history of psychiatry [72]. This phenomenon was defined by Cade [66] as the “pharmacological rehabilitation of lithium.” The first of those clinical trials was a crossover study, placebo-controlled, alternating two weeks of treatment with lithium and two weeks of placebo, which verified Cade’s observations. Published in 1954, the study results confirmed that of the 30 patients with typical mania that had been included, 12 (40%) have shown clinical response to lithium and a worsening of symptoms with placebo. Additionally, another 15 patients have also shown improvement, although it was not possible to differentiate whether such improvement is from a natural remission [73].

Much of the success of the American “pharmacological rehabilitation of lithium” is directly credited to Samuel Gershon (University of Melbourne, Melbourne, Australia), who worked with John Cade before his emigrating to the USA. Gershon convinced many American psychiatrists of the therapeutic efficacy of lithium and published the first article on lithium and mania in the USA [74]. Later, in collaboration with Baron Shopsin, he published another one that, with time, would become the “bible” of pharmacological treatment with lithium salts—“Lithium: its role in psychiatric research and treatment” in 1973 [75].

Steadily, the clinical efficacy of this ion was confirmed. Studies carried out by the group of Schou [76] and Ronald Maggs (Hellingly Hospital at Hailsham, Sussex, UK) [77], under a placebo-controlled method, have demonstrated the superior efficacy of lithium in manic patients. The first American systematic study on the effectiveness of lithium was published in 1966 by Ralph Wharton and Ronald Fieve of New York State Psychiatric Institute. The investigators evaluated 19 patients who had been previously unsuccessfully treated with phenothiazines or electroconvulsive therapy [78]. Two years later, Gordon F. S. Johnson (University of Sydney, Sydney, Australia) and Gershon published the first study of a prospective, controlled double-blinded clinical trial. In their study, the investigators compared lithium and chlorpromazine, a standard treatment for mania, in a total sample of 41 patients. Those authors have obtained a complete or almost complete remission after eight days in 78% of the manic patients treated with 1.5–2 g/day of lithium, compared to only 36% of those who received chlorpromazine [79]. Subsequent studies would also confirm the superiority of lithium against chlorpromazine [80,81,82].

Finally, several highly relevant clinical trials were launched in the late 1960s with public funds from USA Veterans Administration and the National Institutes of Mental Health that dramatically changed the attitude of American physicians towards lithium [83]. In parallel, in the second half of the 1960s, there was a real conflict in the USA between the health authorities and some psychiatrists regarding the therapeutic use of lithium salts, a battle won by the latter. The great champion of this struggle, according to Ayd [84], was Paul H. Blachly, a Professor of Psychiatry at the University of Oregon, who requested that the FDA change its consideration of lithium carbonate, so that it could be given a clinical use. After many bureaucratic obstacles, he received a negative response, despite several appeals to the USA Congress and Senate. Blachly’s final move was to write an aggressive article in the journal *Psychiatric Opinion*, entitled “FDA vs. physician: does the physician have a moral obligation to civil disobedience?” in which he concluded that physicians should have a moral obligation to rebel against health authorities that, as in the case of lithium, exceedingly restricted their prescription rights [85]. Popular pressure and the evidence from the afore-mentioned mentioned clinical trials, finally encouraged the FDA approval of this drug in 1970 (Table 2), 21 years after the publication of Cade’s classic article [61].

### 3.3. Lithium Salts in the Prevention of Manic Episodes’ Recurrence

During the second half of the 1960s, prophylactic effects of lithium in preventing recurrence of manic episodes were also studied. Thus, several authors [22,86,87] found that lithium salts can reduce the number of manic episodes and their duration, as well as increase the duration of the period between episodes. They also found that the episodes have been less severe and that, in cases of relapse, hospitalization times have been shorter. All those facts were confirmed by Schou [88] in an uncontrolled study including 88 patients. The average time until relapse among patients who received a prolonged treatment has been 60 months, whereas before the treatment, the same patients relapsed at 8 months on average. On the other hand, the average annual time of relapse has been 13 weeks before lithium therapy was established, decreasing to 1.5 weeks after treatment. In addition, of the 25 patients who abandoned the treatment, 22 of them relapse in periods ranging from a few days to a year.

Despite many scientific papers supporting lithium prophylactic effect in manic-depressive disorder, the indication for lithium’s antimanic effect, was highly disputed during the second half of the 1960s. Articles entitled “Prophylactic lithium, another therapeutic myth? an examination of the evidence to date” [89] and “A prophylactic myth” [90] illustrate this scenario. The first of them was published in the prestigious journal *The Lancet*, generating great controversy around this issue. This disagreement has been regarded to by some authors as the “Anglo-Danish polemic.” Schou himself would definitively reaffirm his postulates in an article published in 1970 on a study carried out under a rigorous methodology. It was a randomized, placebo-controlled study with a sample of 84 manic-depressive disorder patients, either bipolar (*n* = 50) or depressive monopolar (*n* = 34). Patients were followed for five months, with no relapse in patients in the lithium group and 54% in the placebo group [91]. The relevance of these studies lies on both that they have demonstrated the effectiveness of lithium against manic-depressive episodes and that the study has a genuine innovative methodological design in psychiatric clinical trials, as pointed out by Noguera and Sáiz in 1996 [65].

The efficacy of lithium salts in the prophylaxis of bipolar disorders has definitively been proved in an interesting work by Prien et al. in 1984 [92]. These authors carried out a two-year comparative study in a sample of 117 patients with bipolar disorder, divided into three treatment groups—lithium, imipramine and lithium plus imipramine. The results showed that lithium is more effective in preventing the recurrence of manic episodes (26% in the lithium group, 28% in the lithium plus imipramine group and 53% in the imipramine group), while in the prevention efficacy for depressive episodes all groups are similar (28% of recurrences in the group treated with imipramine, 29% in the lithium group and 22% in the lithium plus imipramine group).

Schou in 1997 provided interesting data on a remarkable variation in the results of studies on the compliance of prophylactic lithium treatments through the last three decades [93]. In this sense, the frequency of therapeutic noncompliance, the main cause of recurrence in prophylactic lithium treatments, was notably higher in the late 1990s than in the 1970s. In addition, the data extracted from clinical trials have also showed a lower effectiveness of this therapy. Schou has attributed these facts to differences in the current profile of manic-depressive patients, whose pathology would be more severe, more atypical (being higher in incidence of schizomanic symptoms) and with greater comorbidity (substance abuse or alcoholism, for example) [93].

### 3.4. Other Clinical Uses of Lithium in Psychiatric Therapeutics

Another clinical usage of lithium salts is associated with its effectiveness in cases of treatment-resistant depression, in combination with antidepressant drugs. This was already revealed in the 1981 classic work of De Montigny et al. [94], who observed that eight patients suffering from unipolar depression and who did not respond to a three-week treatment with tricyclic antidepressants (amitriptyline, imipramine, or doxepin), showed positive therapeutic effect 48 h after adding the administration of lithium. Subsequent studies, larger and better controlled, confirmed those results [94]. In a sample of 75 depressed patients treated for 29 months with antidepressant drugs, 48% of them have been observed to have an additional improvement after lithium salts were added to the existing treatment [95].

Lithium’s potentiating effect of has also been studied more recently in conjunction with antidepressants from the selective serotonin reuptake inhibitors (SSRIs) family such as fluoxetine, yielding even better results than those obtained with tricyclic antidepressants. Ontiveros et al. [96] found that, in a group of 60 patients diagnosed with treatment-resistant depression, 60% have improved after a week when lithium carbonate was added to a regimen with fluoxetine, compared to 57% in patients treated with desipramine when lithium was added. The latter group of patients also shows slow responders.

Although the action mechanism underlying this synergistic effect has not been elucidated, a reinforcement of serotonergic neurotransmission has been hypothesized. In this sense, lithium causes a presynaptic increase in serotonergic function, due to an increase in the reuptake of tryptophan for its conversion into serotonin [46].

Finally, in the late 1970s, many articles assessing the effectiveness of lithium in various mental disorders were published. These covered schizophrenia, substance abuse (including alcoholism), aggressive or impulsive abnormal behaviours, premenstrual syndrome, and so forth. Results, unfortunately, were rather poor.

### 3.5. Conclusions from the “Cinderella of Psychopharmacology”

Despite all its drawbacks and adverse effects from hand tremors to renal toxicity, today, almost seventy years after Cade’s work, lithium salts are the first-choice treatment for the manic phases of bipolar affective disorders, as well as indispensable prophylactic tools in the prevention of cyclic episodes of bipolar disorder. In addition, thanks to the clinical introduction of lithium, the process that has come to be called “The Revolution of Psychopharmacology” was initiated. This means that, for the first time in history, the rational pharmacotherapeutic treatment of mental disorders took place, a process that would be completed with the clinical introduction of chlorpromazine, imipramine and benzodiazepines in the following years [24,97,98,99,100]. But despite these great contributions, lithium is a clear example how an effective drug was unable, in an unwelcoming environment, to find its therapeutic gap. Nathan Kline, one of the great pioneers in psychopharmacology, said in 1968, just when its properties began to be recognized by the scientific community: “Lithium the 20-year old Cinderella of psychopharmacology is at last receiving her sovereign due. Just plain old lithium—discredited by earlier abuse, briefly but dramatically misused as a substitute for precisely the type of patient in cardiac or renal failure for whom it should not be ordinarily used—the modest proclamation of its use for mania and other excitement states in a journal of limited circulation in a remote country that passed almost unnoted” [101].

## 4. Valproate as Mood Stabilizer

### 4.1. The Fortuitous Discovery of Valproate’s Anticonvulsant Activity

Beverly S. Burton in the USA was the first person who synthesized valproic acid in 1881 [102,103]. Valproic acid is 2-propylvaleric acid or *N*-dipropylacetic acid. It is a simple branched chain octacarbon aliphatic molecule [103,104]. It was not thought to be clinically useful because of its poor solubility in water but good solubility in fat [104]. It was only used as an organic solvent at that time.

During World War II, German scientists tried to find substitutes for butter [105] and rediscovered the synthesis of valproate. They did not know of Burton’s synthesis of valproate. After WWII, valproic acid became a popular organic solvent in various industries in Western countries. It was commonly used as a diluent to solubilize other drugs [106].

In 1963, George Carraz and his colleagues working at the Laboratoire Berthier in Grenoble, France, studied the anticonvulsant activity [106] of various khellin compounds [103]. They used valproic acid as a diluent to dissolve the khellins. The results of their study showed that a correlation between anticonvulsant activity and different doses of the tested compound cannot be established but that the diluent has the anticonvulsant activity instead of the khellin derivatives using the pentylenetetrazol convulsion model [104,106]. The investigators realized that all of the solutions contain valproic acid [106,107,108,109]. Therefore, it has to be the effective anticonvulsant, describing an excellent story of pure serendipity [110].

### 4.2. Clinical Drug Trials for Valproate’s Antiepileptic and Mood-Improving Properties

After Carraz and his team members knew that valproic acid had an antiepileptic effect, he synthesized valpromide (Depamide^®^), a derivative of valproic acid [106]. The investigators believed why the concentration of amine compounds such as imipramine or desipramine are higher in the brain because those drugs have an –N–(CH_3_)_2_ or –NH–CH_3_ group, having an obvious lipid solubility. Those drugs can cross the blood-brain barrier easily, resulting in having higher concentration in the brain [106]. In Carraz’ laboratory, the investigators showed that in animals, epileptic convulsions triggered by strychnine can be prevented by valpromide, not by valproate (Depakine^®^), another sodium-based derivative of valproic acid [106].

**Figure 14:**
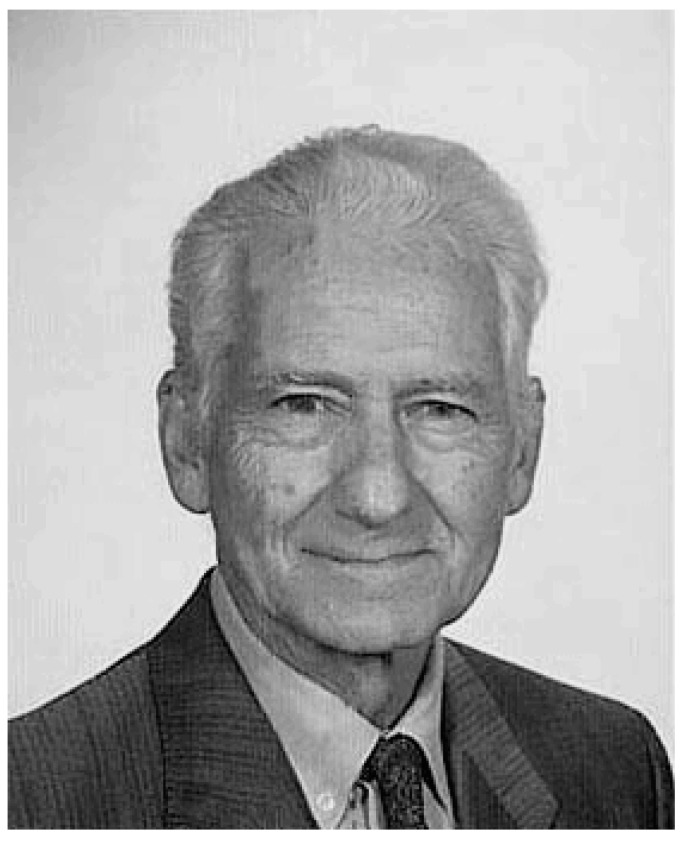
Pierre A. Lambert, French psychiatrist, a pioneer in the field of psychopharmacology. He was a member and president of the prestigious Comité Lyonnais de Recherches Thérapeutiques en Psychiatrie. His role in the conceptualization of mood stabilizers gained great importance in the early stages of the psychopharmacological era.

Carraz collaborated with Sergio Borselli, a psychiatric trainee under Pierre Lambert (Figure 14) at the Psychiatry Hospital of Brittany (Hôpital Psychiatrique de Bassens) in Rhône-Alpes, France [111]. They conducted a series of clinical drug trials in epileptic patients [111,112,113]. As often seen in large asylums in Europe at that time, 10% to 20% of patients were suffering from epilepsy [106]. Borselli and Lambert found that valpromide works as a sedative, particularly when used with other existing anticonvulsants such as phenobarbitone [106]. They also observed that valpromide and valproate have anticonvulsant [114] and psychotropic effects even when administered alone. Lambert described “patients felt more themselves, the mental stickiness, viscosity that had sometimes been there on older agents, was less. We saw the disappearance of tendencies to depression, sometimes even a mild euphoria” [115]. The first report of mood-stabilizing effects of valproate in patients with bipolar disorders appeared in 1966 in the French literature [116]. Besides the French study on the psychotropic benefit of valpromide, Hinderk M. Emrich and associates at the Max Planck Institut für Psychichatrie in Munich, Germany also independently discovered that valpromide has antimanic efficacy [117].

### 4.3. Controlled Clinical Trials Leading to Valproate’s FDA Approval for an Antimanic Indication

Valproate was first introduced as an antiepileptic drug in France in 1967. It has been used in the Netherlands and Germany since 1968, as well as in the United Kingdom since 1973. It became available in the USA in 1983 [104]. There were four oral preparations—valproic acid (Convulex^®^); sodium valproate (Depakene^®^); divalproex sodium (Depokote^®^), an enteric coated compound containing equal proportions of valproic acid and sodium valproate; as well as diavalproex sodium sprinkle capsule (Depokote Sprinkle^®^). The manufacturer, Laboratorie Berthier, had long stopped producing and promoting valpromide as sodium valproate. But it has been sold well in France and abroad [106].

Psychiatrists at the Abbot Pharmaceutical Company, Illinois, USA, owned the patent for divalproex sodium. They were interested in pursuing its antimanic effect. They supported a landmark multicentred, randomized, double-blind, placebo-controlled, parallel group drug trial [118]. This study included 179 acutely manic hospitalized patients meeting for the diagnosis of manic disorder of the Research Diagnostic Criteria [119]. Being considered as one of the most stringent clinical drug trials with the largest number of patients with bipolar disorders in history, the main results of the study showed that divalproex and lithium are more effective than placebo to reduce the symptoms of acute mania and that divalproex is as good as lithium as an antimanic treatment [118]. In 1995, the FDA approved divalproex sodium for its antimanic effects (Table 2) based on the results of this study and other studies [120,121]. The 1994 study by Bowden et al. [118] also proved that lithium is better than placebo in a high-quality placebo-controlled design for the first time with a respectable sample size in this industry-sponsored clinical drug trial, although lithium had already been approved by the FDA in 1970 (see Table 2).

### 4.4. Increased Popularity of Prescribing Divalproex Sodium by American Psychiatrists in the 1990’s

USA FDA approval of divalproex sodium for an antimanic indication was received enthusiastically by psychiatrists in the USA. At that time, divalproex sodium was a major psychiatric product in the USA due to its extensive commercial promotion. Divalproex sodium was often featured besides the AADs (such as risperidone or olanzapine) in symposia sponsored by industry and in supplementary issues of professional journals. From 1994 to 1997, the number of divalproex sodium prescriptions was soared in the USA, whereas that of lithium was declined [122]. For example, 15.5% of adult inpatients received divalproex sodium in 1994 and 34.1% of patients among all public mental health facilities in the State of New York in 1996. Furthermore, 50% of patients with the diagnoses of bipolar disorder and schizoaffective disorder received divalproex in 1996 [122]. By contrast, the number of prescription of lithium was decreased year by year [123]. Both valproic acid and lithium have gained popularity in Latin-American countries. Heenren et al. [124] sent out a 14-item questionnaire to psychiatrists from eight Latin-American countries—Argentina, Colombia, Costa Rica, Ecuador, Mexico, Peru and Venezuela—to study their treatment preferences for patients with bipolar disorder. The authors reported that lithium and valproic acid were by far the top choices among Latin-American psychiatrists when asked about the first mood stabilizer of choice for a patient with bipolar disorder and that with a manic episode, 50% and 41.6%, respectively [124].

The number of prescriptions of divalproex sodium in the USA reached a plateau in late 1990’s and then declined due to the advent of AADs. Like lithium, valproate has been approved as an add-on to increase anti-manic efficacy with olanzapine and risperidone in 2003, quetiapine in 2004, as well as aripiprazole in 2008 (Table 2).

The popularity of valproate prescription was further decreased in the 2000’s because the antimanic and maintenance efficacy of an AAD such as olanzapine are better than those of valproate [125]. At the same time, valproate has also been found to have problematic side effects such as gastrointestinal effects, tremor, sedation, pancreatitis, liver toxicity, weight gain and polycystic ovarian syndrome. Although some treatment guidelines for bipolar disorder suggest that valproate should be avoided during pregnancy due to its teratogenic risk (British Association for Psychopharmacology Guidelines), others, such as the World Federation of Societies of Biological Psychiatry Guidelines recommends close monitoring and risk evaluation in every case. However, in relation to teratogenicity, especially in unplanned pregnancy and with other adverse effects of valproate, such as the risk polycystic ovary syndrome, the benefits should be highlighted often outweigh the risks as patients may over-estimate the risk. Apart from more choice from AADs, valproate prescription has also been hurt by the findings that valproate has proven not to be as good as lithium in anti-suicidal efficacy [126] and maintenance therapy in preventing relapse [127]. Thus, the number of prescription was declined in the decades of 2000s and 2010s compared to 1990s in any country in the world where AADs were available.

## 5. The History of Carbamazepine as an Agent for Bipolar Disorders

### 5.1. Japanese Psychiatrists Discovered Carbamazepine’s Antimanic Properties

Carbamazepine, a tricyclic anticonvulsant compound, was developed in the late 1950s in the laboratories of J. R. Geigy, in Basel, Switzerland [128]. In 1961, Walter Schindler described its synthesis. In 1963, W. Theobad and H. A. Kunz reported its antiepileptic properties. Its efficacy for patients with epilepsy and paroxysmal pain syndromes was demonstrated in Europe in the early 1960’s [128].

In the early 1970’s, lithium was not available in Japan [129]. This led to the use of other agents to treat manic-depressive disorders in Japan [129]. Japanese hospitals were also in the process of institutionalizing with large increasing hospital population following chlorpromazine’s discovery [129,130]. This trend was opposite to what happened elsewhere in the world [130]. In addition, psychiatry was neuropsychiatrically oriented and psychiatrists instead of neurologists treated most patients in Japan [129,130]. As a result, carbamazepine was widely used in Japanese hospitals following its release as an anticonvulsant during the 1960’s [129].

**Figure 15:**
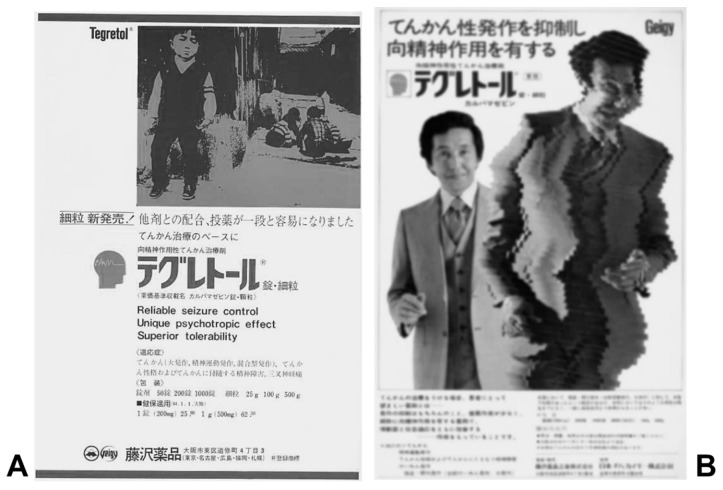
Carbamazepine (Tegretol^®^) advertisements in the Japanese medical press during the 1970s, which emphasized its anticonvulsant and sedative effects. Ads published in the journal *Psychiatria et Neurologia Japonica* in (**A**) 1970 and (**B**) 1978.

The sedative properties of carbamazepine (Figure 15) led to its use for manic patients instead of other sedatives such as the barbiturates. In the valpromide story for treating manic patients [114], carbamazepine was applied conditions by Japanese psychiatrists exclusively to patients with epileptic or manic [106]. Takezaki and Hanaoka [129] in Tottori, Japan reported their first clinical series of drug trials for manic-depressive patients with carbamazepine. They reported their results in a Japanese journal in 1971 [129]. After the report, many Japanese researchers, especially from the Teruo Okuma group, continued to demonstrate carbamazepine’s advantage in treating patients with manic-depressive illness [131,132,133,134].

### 5.2. Information of Carbamazepine’s Research Results Spread to the West from Japan

In a personal reflection, Okuma in 2000 stated that Western psychiatrists were not impressed when the Japanese reported about carbamazepine’s psychotropic effectiveness was published in English [135]. It stated that carbamazepine has similar efficacy compared to chlorpromazine, which was discovered in 1952 [136,137]. The major criticism of the 1979 article [135] was that there was no real evidence of efficacy of carbamazepine at all. The chlorpromazine dose (250 mg/day) in their study was considered inadequate [134]. The reason this research report was not accepted was that a 250 mg dose of chlorpromazine was thought to be indistinguishable from placebo at a time when Western psychiatrists were using antipsychotic mega-doses [106,134]. The research protocol was similar to the one used to investigate lithium’s efficacy but those outcomes were not contested [106]. The results of the antimanic efficacy of carbamazepine and lithium are comparable [138,139]. Even carbamazepine did not come into wide use in the West until 1878 when its antimanic effects were described by the American psychiatrists James Ballenger and Robert Post at the USA National Institute of Mental Health [140]. By the time it became more widely used, it was clear that carbamazepine had imported psychotropic properties. Carbamazepine was used in Japan for a wide range of mental conditions (Figure 15). It was useful in stabilizing aggressive outbursts in young men [103]. Later, the use of carbamazepine for the management of episodic dyscontrol syndrome [103,141] was described in the Western literature [106].

### 5.3. Current Status of Carbamazepine for the Treatment of Bipolar Disorders in the USA

Carbamazapine has been observed to induce blood dyscrasias delayed its use in North America [106], even though this serious adverse side effect was rare. USA FDA approved carbamazepine as an antiepileptic agent for adults in 1974 and for children older than 6 years of age in 1978. The FDA allowed its use for all without any age limitations in 1987. In Latin-America, there is a high degree of polypharmacy in the treatment of bipolar disorder, with anticonvulsants being the most commonly prescribed medication. Among anticonvulsants, valproic acid was the most popular, followed by clonazepam and then carbamazepine [142]. As shown in Table 2, slow-release carbamazepine received FDA’s approval for treating mania in 2004 but its use has never been expanded to other indications for other clinical phases of bipolar disorder.

Oxcarbazepine has a chemical structure with a keto group instead of a double-bond between two carbons in the central ring of carbamazepine. Those structures have also been shown to have antimanic efficacy in Europe [143]. Oxcarbazepine has a favourable side effect profile producing less blood dyscrasias and less drug-drug interactions. This drug was introduced as an antiepileptic to the USA from Europe under the trade name of Trileptal^®^ in 2000. To date, the pharmaceutical industry is not interested in developing carbamazepine or oxcarbazepine for treating other phases of bipolar disorder.

## 6. New Antiepileptics in Bipolar Disorders

Despite the progress accomplished, treating bipolar disorder with lithium, valproate and carbamazepine involves four major loopholes:

- Many patients are resistant to conventional treatment.
- Good number of patients present themselves with tolerability problems, due to the frequent adverse effects of those drugs.
- The depressive phase still constitutes a serious problem, since lithium, carbamazepine and valproate are more effective in relieving symptoms in the manic than the depressive phase.
- Rapid cycling and mixed mania (dysphoric) respond poorly to treatment, etc. [144,145,146].

Although carbamazepine was already used in Europe as a substitute for lithium or in combination in resistant cases, the commercial success of valproate was the ultimate reason that stimulated the evaluation of the new anticonvulsant agents in treating bipolar disorders, particularly when it has been demonstrated in the field of epilepsy that they entailed better profile of side effects, interactions, toxicity and teratogenicity [147]. With those premises and significant financial support from the pharmaceutical industry, those new antiepileptics are aimed to displace lithium and second-generation anticonvulsants in treating bipolar disorder, a pathology at the entrance door to the emerging juicy market of psychiatric disorders.

In the individual analysis of the use of these new antiepileptic drugs in bipolar disorder, lamotrigine has been the most contrasted agent, both from the clinical and safety perspectives [148,149]. Lamotrigine has been licensed for the indication in preventing relapse of having future depressive episodes in patients with bipolar disorder.

### 6.1. Lamotrigine in the Prevention of Depressive Episodes in Bipolar Disorder

Lamotrigine was the third of the so-called new generation of antiepileptics to be marketed, after vigabatrin and felbamate. But those last two drugs were not studied as mood stabilizers because after their commercialization as antiepileptic drugs, significant idiosyncratic side effects were discovered, such as visual field loss or aplastic anaemia, limiting their use to special cases of epilepsy (Lennox-Gastaut syndrome, for example). In the case of lamotrigine, its side effects profile is more favourable in general than that of classic antiepileptic drugs, despite the feared of infrequent Stevens-Johnson syndrome.

Lamotrigine is a phenyltriazine that was synthesized in the early 1980s at Wellcome Research Laboratories (Beckenham, Kent, England) as part of a program to develop new antiepileptic agents that could be better tolerated than those available at that time [150]. This program was based on the mid-1960s hypothesis that folates had proconvulsant properties, so that antiepileptics were thought to exert folic acid antagonistic effects [151]. Taking pyrimethamine as a reference, a substance developed in 1950 as antimalarial (for the treatment and prevention of malaria), a series of structurally analogous phenyltriazines were synthesized to improve its anticonvulsant activity. One of these compounds, BW288U, presented a good anticonvulsant activity but a low antifolate activity, which they tried to optimize. As a result, lamotrigine was obtained, a compound with great anticonvulsant activity but weak dihydrofolate reductase inhibitory power [152]. Phase I clinical trials started soon, showing excellent pharmacological properties. Lamotrigine was finally approved in 1990 in Ireland for use in epilepsy. In 1994, USA FDA also approved its use in a combined treatment for this neurological disorder. Similarly, in those initial studies, Smith et al. [153] observed that this agent improves mood and communication skills in treated epileptic patients.

The first author who produced scientific data on the efficacy of lamotrigine in bipolar disorder was Richard H. Weisler (University of North Carolina Chapel Hill School of Medicine at Chapel Hill, North Carolina, USA). Having attended several international conferences, Weisler knew this molecule before it was approved in the USA and anticipated its possible use in psychiatry. After importing the drug from Europe and requesting authorization from the FDA for compassionate use in 1993, Weisler used lamotrigine to treat two long-term bipolar patients—a 43-year-old white male patient with bipolar II disorder and rapid cycling, as well as a 77-year-old female patient with bipolar I mood disorder with predominance of major depressive episodes and several suicide attempts. At that time, those two patients were refractory to all available pharmacological treatments—lithium, carbamazepine, clonazepam, valproate, 11 different antidepressants (including some of those only being available in Europe then), buspirone, levothyroxine and verapamil. The treatment regimen included, in addition to lamotrigine, other pharmacological agents such as lithium carbonate, bupropion and levothyroxine, in one case and only levothyroxine, in the other case. Both patients showed an evidence of clinical improvement after several weeks, a rapid normalization of social and family relationships and one of them even resumed working activities. Weisler’s group presented the results obtained with those two patients at the American Psychiatric Association’s annual meeting held in 1994 in Philadelphia [154] and defended their use based on its action mechanisms (potent anti- kindling effect, sodium channel blockade and anti-glutamatergic activity). In the following two years, several more articles were published about diverse clinical cases, such as refractory bipolar patients, patients with overt depressive episodes, and/or rapid cycling [150].

In 1994, after the presentation of those clinical cases, the company marketing lamotrigine, Burroughs Wellcome, brought together in its research facilities in Beckenham (England) a series of international experts in bipolar disorder to address the possibility of developing of a research program on lamotrigine to treat this psychiatric disorder. Initially, they agreed to start an open, multi-centre clinical trial (five international centres) with a sample of 75 patients in any active phase of the disorder, either type I or type II and a follow-up of 12 months, treated with lamotrigine in monotherapy or in combination treatment. The results revealed that an 81% response rate and a 74% decrease in the Young Mania Rating Scale (YMRS) score are found in patients in the manic/hypomanic phase and that a 48% response rate and a 42% decrease in the Hamilton Depression Rating Scale (HDRS) score are also found in those in the depressive phase. Secondary analyses have shown additional benefits in other aspects related to the depressive episode and good tolerability with the most common adverse effects being dizziness, tremors, drowsiness, headaches, nausea and rash [155].

Given the positive results of this open study, GlaxoSmithKline launched between 1996 and 2001 an ambitious program of a controlled double-blind phase III trials and enrolled 2400 patients from four continents—5 trials in acute episode of bipolar depression, 2 in acute episode of mania, 2 in the prevention of manic or depressive episode and 2 in rapid cycling patients [150]. Unlike typical antipsychotics being more effective in the control of mania than depression, lamotrigine is especially effective in patients with a predominance of depressive phases. The results of the first double-blind, placebo-controlled clinical trial confirmed its effectiveness in bipolar depression at doses between 50 and 200 mg/day [156], although the improvement in the Hamilton 17-item Depression Scale, the main efficacy variable, is not significant. Similar results were obtained in the other four trials. These poor results might be due to the need to slowly increase the dose of lamotrigine (up to 10 weeks to reach the final dose) to avoid the appearance of rash [157]. Subsequently, a meta-analysis considering all trials would confirm a significant, albeit small, efficacy of the treatment with lamotrigine [158]. By contrast, in double-blind clinical trials with patients in the manic phase, lamotrigine (50 mg) yielded negative results [159], presenting no statistical differences with placebo and lower efficacy than lithium. But in those studies, the risk of shift from mania to depression is not more than to placebo.

Yet, the best results with lamotrigine were obtained in studies of relapse prophylaxis in patients with bipolar disorder shown in two randomized double-blind trials which compared the efficacy of lamotrigine with that of lithium and placebo over 18 months. As a result, the USA FDA approved its use for this indication. Altogether (*n* = 1300 patients), both trials concluded that lithium is superior to lamotrigine and placebo in the prevention of manic phases and that lamotrigine is superior to lithium and placebo in the prevention of depressive phases [148,149]. The combination of data from both trials also showed that lamotrigine is effective in the prevention of mania, although the results is not as robust as that with lithium [160]. These findings confirmed the characteristically antidepressant profile of lamotrigine as a mood stabilizer, as opposed to other drugs available at the time and obtained its approval by USA FDA in 2003 (Table 2). In addition, they suggested the possibility to combine lamotrigine with lithium to obtain more symmetrical results in relapse prevention [161].

The effectiveness of lamotrigine in rapid cycling patients was also suggested, although data were based on a placebo-controlled clinical trial, is only positive in bipolar type 2 [162]. Finally, it was also confirmed that the combination of lithium and lamotrigine is safe and useful in refractory bipolar depression [163,164] and that the combination with valproate or carbamazepine is also possible, although implying more interaction risks [161].

### 6.2. Other Modern Antiepileptic Agents

Gabapentin was one of the first third-generation antiepileptic drugs to be studied for bipolar disorder and as almost all of them, it was initially evaluated for the treatment of mania. Although most open studies have positive outcomes [165] but the two controlled clinical trials produce negative results [166,167] and raised considerable doubts about its efficacy. In the first one [166], lamotrigine shows better outcomes than gabapentin. In the second one, surprisingly, placebo is better than gabapentin in manic patients treated with lithium [167]. However, gabapentin could be an interesting drug as an enhancer of other mood stabilizers in cases in patient with a high anxiety component [168]. This is especially relevant at present, since many authors advocate combined treatment to prevent the natural disease progression towards a shortening of remission periods [169].

The history of topiramate shows paradigmatically how research on potentially anticonvulsant drugs is evolving. Topiramate was discovered in 1979 by Bruce E. Maryanoff and Joseph F. Gardocki through their research work at McNeil Pharmaceuticals (Fort Washington, PA, USA), a subsidiary of Johnson & Johnson Corporation. It was initially synthesized as a hypoglycaemic agent. In fact, topiramate is a sugar, a derivative from fructose. After having known its potential efficacy in diabetes being limited, the investigators studied topiramate in various animal models of different diseases, proving its anticonvulsant efficacy in rats. The preclinical research showed that the presumed antidiabetic was a potent antiepileptic. Some open studies suggested that it could be an effective drug as an add-on therapy in bipolar patients with partial response to other drugs [170] and a monotherapy [171]. But placebo-controlled clinical trials in manic patients cannot prove its efficacy [172], despite that several controlled trials of high methodological quality were carried out.

As for tiagabine, little relevant data exist to show its effect in bipolar disorder, although a study conducted in the Stanley Foundation Bipolar Network indicated that its mood stabilizing potential is scarce or null, together with a certain risk of seizure induction [173].

As mentioned before, oxcarbazepine, a 10-keta analogue of carbamazepine, was developed in the 1970s as an improved alternative to carbamazepine. In addition to the initial studies from the 1980s, recent data exist from experiments using oxcarbazepine to treat bipolar patients. Case series with positive results have been reported [174]. In addition, an open study with 12 patients receiving oxcarbazepine has been published, showing good results in participants with mild or moderate forms of mania, worsening in symptoms after discontinuing the drug and becoming improved again after resuming treatment [175]. In any case, the APA advises the use of oxcarbazepine as a reasonable option in treating bipolar patients who do not respond to established treatments but more clinical trials are needed to confirm its potential as mood stabilizer in the maintenance treatment. Licarbazepine, an active metabolite of oxcarbazepine, was also evaluated in controlled clinical trials for manic episodes in bipolar disorder patients, with unsatisfactory results [176].

With respect to levetiracetam, pregabalin, retigabine, or zonisamide, the data published from patients with bipolar disorder are limited, although their properties are being assessed as mood stabilizers.

## 7. Atypical Antipsychotic Agents in Bipolar Disorders

The delay in the action onset of classical mood stabilizers in the treatment of symptomatic acute manic symptoms enabled the study with the use of antipsychotic drugs in this disorder [177]. Chlorpromazine was the only traditional antipsychotic drug (or first-generation antipsychotic drug, FGA) approved by USA FDA in 1973 for the treatment of manic episodes in bipolar disorder. Several clinical trials contrasted the efficacy of chlorpromazine in acute mania, both in monotherapy [83,178] and in combined treatment with lithium salts [179]. Nevertheless, its use in bipolar disorder was limited due to its poor tolerability, causing sedation and hypotension and the advent of new atypical antipsychotic drugs (AADs, or second-generation antipsychotic drugs, SGAs). The clinical introduction of risperidone on the USA market in 1993 to treat patients with schizophrenia heralds the advent of AADs and the clinical introduction of those agents reinvigorated the interest in therapeutic research on the different phases of bipolar disease [180]. In addition, its wide use in schizophrenia provided contrasted data regarding SGAs’ superior safety over FGAs, especially with respect to extrapyramidal effects.

Besides, the unique biochemical properties of AADs (clozapine, olanzapine, risperidone, quetiapine, ziprasidone, aripiprazole, asenapine and cariprazine), mainly their ability to antagonize dopamine receptors, together with other peculiarities of their pharmacological receptor profile and their effects on multiple neurotransmission systems [181] also fostered their study as potential antimanic agents [182]. In addition to dopamine D_2_ antagonist effects, AADs block 5-HT_2_ serotonergic receptors, basically 5-HT_2A_ and 5-HT_2C_ and some of them, such as aripiprazole, ziprasidone and asenapine, are 5-HT_1A_ receptor partial agonists [181], which could result in an enhancement of noradrenergic and dopaminergic neurotransmission [183]. Although the exact mechanisms of AADs in the treatment of mania remain unclear, it seems obvious that this type of drugs have a specific antimanic effect regardless of the presence of comorbid psychosis or the degree of sedation induced by the agents [2].

Olanzapine was the first atypical antipsychotic drug to have been licensed for the indication of treating acute mania. Eli Lilly (Indianapolis, IN, USA) sponsored six double-blind studies published between 1999 and 2003, to evaluate olanzapine’s efficacy for treating mania or mixed episodes compared to that of placebo, divalproex and haloperidol. The first of those was a placebo-controlled trial [184] with a three-week follow-up period. The study results showed a mean reduction in the YMRS score of 10.26 points in the olanzapine group versus 4.88 points in the placebo group and the percentage of patients being responders in olanzapine vs. placebo were 48.6% vs. 24.2%, respectively. The mean modal dose of olanzapine 14.9 mg/day, is superior to placebo in treating acute symptoms of mania in this trial. Subsequent studies confirmed the efficacy of olanzapine in acute mania, with an efficacy superior to valproate and lithium [185]. As a consequence, olanzapine was the first AAD approved by the USA FDA to treat manic episode of bipolar disorder in 2000 (Table 2).

In any case, the antimanic efficacy of AADs was later revealed by a series of randomized, placebo-controlled studies [12,186,187,188,189,190], suggesting that all AADs, as a pharmaceutic group, have a class-effect in treating mania. Almost all AADs got approval to treat mania one by one—risperidone in 2003; quetiapine, ziprasidone and aripiprazole in 2004; asenapine in 2009; as well as cariprazine in 2015 were approved by the USA FDA as monotherapic agents for the antimanic indication (Table 2). In addition, some of them were also approved for the prevention of relapse in patients with bipolar disorder who in a manic episode had already responded to the treatment with those antipsychotics (Table 2). At present, AADs are the first line of treatment for the manic phases of bipolar disorder [191].

Various AADs expanded their indications to treat all three clinical phases of bipolar disorder. Seemingly most AADs, if not all, can get the indication for relapse prevention if they are combined with the use of lithium or valproate (Table 2). Finally, olanzapine (always in combination with fluoxetine) in 2003, quetiapine in 2008 and lurasidone in 2013 have been approved for the treatment of depressive episodes in bipolar disorder. This may be the reason why, recently, the number of scientific publications on bipolar disorder [9] and AADs have suddenly and exponentially increased in some countries of Asia and Europe and in Australia [192,193,194,195,196].

The treatment of bipolar depression is more complex than the treatment of manic episodes. It requires different therapeutic approaches from unipolar depression [197]. Also, the scientific evidence on the role of AADs is smaller and, in some cases, the results of meta-analyses have given rise to inconsistent conclusions [198]. The antidepressant properties of some AADs could be due to their ability to modulate three monoaminergic systems (noradrenergic, serotoninergic and dopaminergic) involved in the pathophysiology of depression [199]. In the case of quetiapine, its active metabolite norquetiapine helps serotonergic transmission, acting as a partial agonist of 5-HT_1A_ receptors, in addition to working as a potent inhibitor of the noradrenaline transporter, which increases noradrenergic functionality [200,201].

Although olanzapine is approved by USA FDA in the treatment of bipolar depression, that is the case only in combination therapy with fluoxetine. The first AAD to obtain approval from USA FDA for the treatment of bipolar depression in monotherapy was quetiapine, based on a clinical trial program developed by AstraZeneca (London, UK) and known under the acronyms BOLDER (BipOLar DEpRession) and EMBOLDEN (Efficacy of Monotherapy Seroquel in BipOLAR DEpressioN). The BOLDER studies, placebo-controlled and 8 weeks long, confirmed that quetiapine at daily doses of 300–600 mg is superior to placebo in the total reduction of depressive symptoms according to the Montgomery-Asberg Depression Rating Scale. (MADRS) and that the improvement occurred from the first week in all the main symptoms of depression. Likewise, quality of life and anxiety are improved to a greater extent with quetiapine and this improvement was independent of the presence or absence of sedation [202,203]. The efficacy of quetiapine at a 300 mg/day dose is similar to that of 600 mg/day, so both doses have been approved for the treatment of bipolar depression. With respect to EMBOLDEN trials, an assessment was performed at week 8 and patients who met specific response criteria entered a 52-week treatment extension phase. In the EMBOLDEN I study, quetiapine at 600 mg/day (but not quetiapine at 300 mg/day) is superior to lithium in reducing the MADRS score at week 8 [204], while in the EMBOLDEN II study, quetiapine, at doses of 300 and 600 mg/day, reduces the total score of the MADRS scale more than the SSRI antidepressant paroxetine at dose of 20 mg/day. In addition, paroxetine does not differ from placebo [205]. In those studies, in acute episodes of bipolar depression, quetiapine achieves higher and earlier response and remission rates than placebo and it does so from the first or second week.

This decision to pursue clinical drug trial of lurasidone for patients with depressive episode in bipolar disorder was influenced by three relevant factors [206] because lurasidone has:

- special pharmacological profile [207] of receptor affinity (mainly as a 5-HT_1A_ receptor agonist and a 5-HT_7_ receptor antagonist),
- efficacy in animal models of acute and chronic depression [208],
- shown favourable improvement in depressive mood in previous efficacy studies in schizophrenia [209].

Lurasidone is the first AAD whose efficacy in bipolar disorder was studied in the treatment of the depressive phase exclusively. The investigators observed that in patients treated with lurasidone for six weeks, the mean MADRS score is decreased more than those in the control group. Therefore, the pharmaceutical company Sunovion Pharmaceuticals, Inc. (Marlborough, MA, USA) launched in 2009 a clinical trial program called PREVAIL (Program to Evaluate the Antidepressant Impact of Lurasidone), with the aim to evaluate the efficacy and safety of this AAD both in monotherapy and in combination treatment with lithium or valproate, in adult patients diagnosed with bipolar depression according to clinical criteria of *DSM-IV-TR* [199,206]. The positive results of those trials and an open six-month long-term follow-up study allowed USA FDA approval of this agent in June 2013, for the treatment of depressive episodes of bipolar I patients.

## 8. Conclusions

The definition of a “mood stabilizer” is not clear. It means different things to different people (APA, 2002). According to one specific definition, it is a drug that has both antimanic and antidepressant effects on both mood states [210,211]. In conservative definition, it means a drug that has therapeutic efficacy in 2 of 3 different mental states, as shown in Table 2 [212]. But in a broader definition, it means a drug that: (A) is effective in 1 of any 3 phases as an antimanic, antidepressant, or for maintenance/prophylaxis; (B) does not worsen any mood state; and (C) does not have the potential of switching the patient from one to the other mood state [213,214]. According to this broad definition, tricyclic antidepressants do not belong to this category to treat patients with bipolar disorders. The results of meta-analysis data from clinical trials has shown that the rates of mania induced by tricyclics, SSRIs and placebo are 11.2%, 3.7% and 4.2%, respectively [215]. Venlafaxine and paroxetine have been found to have beneficial psychotropic properties. However, venlafaxine, instead of paroxetine, is noted to have a higher mood switch rate compared to paroxetine, 13% vs. 3% [216]. Valproic acid and carbamazepine have never been identified to switch the mood of bipolar patients from a depressive to a manic phase.

The American Psychiatric Association has discouraged the use of the term “mood stabilizer” [143]. It has never been officially used by USA FDA for a drug approved to treat patients with bipolar disorders (Table 2). Not to further invite confusion, we suggest that one should avoid the term “mood stabilizer” as much as possible even though no simple substitute term is available.

In this paper, we have used the USA FDA approval status as the milestone for describing drugs for the treatment of bipolar disorders. Approval by USA FDA frequently means a drug’s marketability not only in the USA but also all over the world. As shown in Table 2, only three AADs (olanzapine, quetiapine and lurasidone) have been approved by USA FDA for the treatment of the depressive phase of bipolar disorder and only two (olanzapine and quetiapine) for all three phases of “mood states.” But olanzapine needs to be combined with fluoxetine, an antidepressant, for the treatment of the depressive phase of bipolar disorder [217]. Lithium and valproate have been approved by USA FDA for antimanic indications in 1975 and 1995, respectively. Since 2000, olanzapine, risperidone, quetiapine, ziprasidone, aripiprazole, asenapine and cariprazine have been approved for antimanic indication. Currently the AAD market is saturated with antimanic agents as listed in Table 2. No more motivation exists for a pharmaceutical company to invest money to develop oxcarbazepine or another antiepileptics for an antimanic indication. Hence, those drugs are most likely to remain off-label use for antimanic effects.

No shortage of available antimanic agents is shown on the market (Table 2). For other antiepileptic drugs, only lamotrigine has been approved by USA FDA for maintenance therapy. But lamotrigine or similar drugs, rather than valproic acid or carbamazepine, continues to draw attention in the treatment of patients with bipolar disorders. Their rare characteristic is their efficacy for both poles of bipolar depression.

### Historical Implications at the Socio-Sanitary Level of the Clinical Introduction of the First Mood Stabilizers

The direct and delayed consequences of the introduction of the first psychotropic drugs in the 1950s have been multiple and lithium salts played a fundamental role [30,63,97,218,219]. At the purely scientific level, they favoured the postulate of the first biological hypotheses on the genesis of mental illnesses, giving birth to the so-called “biological psychiatry.” At the nosological level, the introduction of those drugs contributed to design new, much narrower diagnostic criteria, characterized, to a large extent, by a predictable and homogeneous response to treatment [220]. Clinical research methodology also benefited from the appearance of those drugs, specially lithium salts, with the rise of multicentre, double-blind, cross-over, randomized clinical trials, etc., as well as the development of a large number of generic and specific tests for the assessment of changes in psychopathological traits. Lithium played a decisive role in this whole process [62].

From the historical perspective, another relevant contribution of the clinical introduction of lithium and the FGAs lays at the health care level, giving rise to the progressive phenomenon of “deinstitutionalization” in psychiatry. This fact mitigated the stigma component that had accompanied psychiatric assistance. In this sense, the FGAs not only enabled patients to leave psychiatric hospitals but also helped their re-socialization. Some data show the impact that the introduction of these agents had. During the first half of the 20th century, the number of patients admitted to psychiatric hospitals in the USA was alarmingly increased, from 150,000 to 500,000 patients. But from 1955, the year in which certain psychopharmaceuticals (FGAs and lithium salts, particularly) began to be used on a massive scale, the rate of hospitalizations was reversed and in 1975 the number of patients was dropped to 200,000 [221]. Another example comes from the economic benefits that lithium brought to the community, which have been estimated, in the USA, at USA $145 billion dollars from its USA FDA approval in 1970 until 1994, mainly due to savings in hospitalization costs [222].

Even today, almost 70 years after Cade’s work, lithium salts, applying the precautions associated with a drug of moderate toxic character (basically the control of lithium), constitute the first-choice treatment for the manic phases of affective bipolar disorders, as well as indispensable prophylactic tools in the prevention of cyclic episodes of manic-depressive illness. Even the “black history of lithium toxicity” should be revised, as proposed by Moncrieff [54], since, while the rate of deaths directly attributable to lithium stands at 14 per million prescriptions, this ratio rises to 50 in the case of dothiepin, 46.5 in the case of amitriptyline or 11.1 with clomipramine. In any case, the importance and transcendence of lithium in psychiatric therapeutics is reflected on the enormous amount of scientific publications on this agent, which reached 9500 in the 33 years after the classic work of Cade [223].

Currently, psychiatrists dispose of a therapeutic arsenal of 13 different drugs approved by regulatory agencies to address the different acute episodes of bipolar disorder and the prevention of relapse. Although the process was started 70 years ago by John Cade with the discovery of lithium salts antimanic effect has not yet concluded and there are broad aspects to be improved, the pharmacological treatment of bipolar disorder has established itself as a substantial part of the history of psychopharmacology. In any case, the scientific evidence accumulated over the last decade appears to confirm that there is no unitary biochemical mechanism to explain the effect of all the substances currently available for the treatment of bipolar disorder. In fact, research in this field is advancing in other directions, with the aim of finding new loci of drugs’ action, such as the role of neurotrophic factors (neurotrophins and BDNF), corticotropin-releasing hormone (CRH) and glucocorticoids, excitatory amino acids and NMDA (*N*-methyl-d-aspartate) receptor, as well as other possible targets (opioid system, interleukins, P substance, somatostatin, neuropeptide Y, melatonin, nitric oxide and so on). One of the most important objectives of the current neuroscience is the discovery of the molecular mechanisms responsible for intraneuronal communication. Detailed knowledge of these mechanisms could provide us, in the future, with much more specific and safer pharmacological tools for treating bipolar disorder. But this story continues to be written...

## Figures and Tables

**Table 1:** Key milestones in the history of the pharmacological treatment of bipolar disorder.

Year	Important Events
1817	Isolation of lithium (Arwedson and Berzelius)
1832	Synthesis of chloral hydrate (von Liebig)
1857	Introduction of bromides as hypnotic-sedatives (Locock)
1863	Synthesis of barbiturates (von Baeyer)
1868	Description of the sedative-hypnotic properties of *Solanaceae* alkaloids (Schroff)
1869	Use of chloral hydrate as a hypnotic (Liebreich)
1870	Use of chloral hydrate in manic patients (Elstun)
1880	Isolation of hyoscine or scopolamine (Ladenburg)
1881	Synthesis of valproic acid (Burton)
1903	Introduction of barbital in medicine (Fisher and von Mehring)
1912	Marketing of phenobarbital
1915	Introduction of barbiturate sleep cures (Epifanio)
1920	Application of morphine and scopolamine “sleep cures” (Klaesi)
1943	Introduction of promethazine in psychiatry, for the treatment of manic symptoms (Daumézon)
1949	Introduction of lithium in the management of manic and schizophrenic disorders (Cade)
1954	First controlled clinical trial with lithium in manic patients (Schou)
1957	II World Congress of Psychiatry (Zurich): first classification of psychotropic drugs (Delay) International Symposium on Psychotropic Drugs (Milan): first specific scientific meeting on psychotropic drugs
1958	Foundation of the Collegium Internationale Neuropsychopharmacologicum
1960	Confirmation of the prophylactic effect of lithium salts in manic episodes (Schou)
1961	Synthesis of carbamazepine (Schindler) Foundation of the American College of Neuropsychopharmacology
1963	Discovery of the anticonvulsant effect of valproic acid (Carraz)
1966	First data on the antimanic effect of valproic acid (Lambert) First systematic study on the effectiveness of lithium in the USA (Wharton and Fieve)
1970	Demonstration of the prophylactic properties of lithium in manic-depressive psychosis (Schou) Approval of the clinical use of lithium (USA FDA)
1971	Use of carbamazepine as mood regulator (Takezaki and Hanaoka)
1973	Approval of chlorpromazine in the treatment of manic episodes (USA FDA) Publication of the book *Lithium: its role in psychiatric research and treatment* (Gershon and Yuwiler)
1978	Approval of lithium salts for the prevention of manic/depressive episodes (USA FDA) First study about the antimanic effects of carbamazepine in the West (Ballenger and Post)
1994	First controlled trial with divalproex in mania (Bowden) First publication on the efficacy of lamotrigine in bipolar disorder (Weisler)
1995	Approval of valproic acid as an antimanic drug (USA FDA)
1999	First controlled trial with olanzapine in manic episodes (Tohen)
2000	Approval of olanzapine in bipolar disorder (USA FDA)
2003	Approval of risperidone in bipolar disorder (USA FDA) Approval of the combination olanzapine-fluoxetine in depressive episodes of bipolar disorder (USA FDA) First controlled trials of lamotrigine on relapse prophylaxis in bipolar disorder (Bowden and Calabrese) Approval of lamotrigine for the prevention of depressive episodes of bipolar disorder (USA FDA)
2004	Approval of quetiapine, zipresidone and aripiprazole in bipolar disorder (USA FDA) Approval of olanzapine for the prevention of new episodes of bipolar disorder (USA FDA)
2005	Approval of aripiprazole for the prevention of new episodes of bipolar disorder (USA FDA)
2007	Approval quetiapine for the prevention of new episodes of bipolar disorder (USA FDA)
2008	Approval of quetiapine in depressive episodes of bipolar disorder (USA FDA)
2009	Approval of asenapine in bipolar disorder (USA FDA) Approval of risperidone and ziprasidone for the prevention of new episodes of bipolar disorder (USA FDA)
2013	Approval of lurasidone in depressive episodes of bipolar disorder (USA FDA)
2015	Approval of cariprazine in bipolar disorder (USA FDA)

**Table 2:** Drugs approved by the Food and Drug Administration of the United States (USA FDA) for treatment of patients with bipolar disorder.

Drug	Mania	Maintenance/Prevention	Bipolar Depression
Lithium	1970	1978	
Valproate	1995		
Carbamazepine	2004 ^a^		
Lamotrigine		2003	
Chlorpromazine	1973		
Olanzapine	2000, 2003 ^b^	2004	2003 ^c^
Risperidone	2003, 2003 ^b^	2009 ^d,e^	
Quetiapine	2003, 2004 ^a,^^b^, 2008 ^f^	2007 ^g^	2008 ^f^
Ziprasidone	2004, 2004 ^b^	2009 ^e,g^	
Aripiprazole	2004 ^h^, 2004 ^b^	2005 ^e^, 2017 ^d^	
Asenapine	2009		
Cariprazine	2015		
Lurasidone			2013, 2018 ^i^

^a^ Slow-release formulation; ^b^ Adding lithium or valproate increases antimanic efficacy; ^c^ Requiring combination with fluoxetine; ^d^ Long-active injectable form; ^e^ Efficacy mostly for preventing future manic episodes; ^f^ Sustained-release galenic form; ^g^ Requiring the addition of lithium or valproate; ^h^ Having data up to three months; ^i^ In paediatric patients 10 to 17 years old.

## Abbreviations

AAD	Atypical Antipsychotic Drug
APA	American Psychiatric Association
BOLDER	BipOLar DEpRession
DSM	Diagnostic and Statistical Manual of Mental Disorders
ECA	Epidemiological Catchment Area Study
EMBOLDEN	Efficacy of Monotherapy Seroquel in BipOLAR DEpressioN
FDA	Food and Drugs Administration
FGA	First-Generation Antipsychotic Drug
HDRS	Hamilton Depression Rating Scale
MADRS	Montgomery-Asberg Depression Rating Scale
NCS	National Comorbidity Survey
POW	Prisoner of War
PREVAIL	Program to Evaluate the Antidepressant Impact of Lurasidone
SGA	Second-Generation Antipsychotic Drug
SSRI	Selective Serotonin Reuptake Inhibitors
UK	United Kingdom
USA	United States of America
WHO	World Health Organization
WWII	World War II
YMRS	Young Mania Rating Scale
